# Clinical outcomes of Ahmed glaucoma valve implantation without fixation of a plate: The free plate technique

**DOI:** 10.1371/journal.pone.0241886

**Published:** 2020-11-06

**Authors:** Han Min Lee, Kee Sup Park, Yoo Young Jeon, Woo Jin Kim, Nam Ho Lee, Kyoung Nam Kim, Chang-sik Kim

**Affiliations:** 1 Department of Ophthalmology, Chungnam National University Hospital, Daejeon, Republic of Korea; 2 Noonsarang Eye Clinic, Daejeon, Republic of Korea; 3 Mindeulle Eye Clinic, Boeun, Republic of Korea; Pusat Perubatan Universiti Kebangsaan Malaysia, MALAYSIA

## Abstract

**Objective:**

This study compared surgical outcomes between free plate Ahmed glaucoma valve (FPAGV) implantation without plate fixation and conventional Ahmed glaucoma valve (CAGV) implantation with plate fixation.

**Methods:**

A retrospective, comparative case series study. Patients with refractory glaucoma who underwent FPAGV or CAGV implantation and were followed >1 year were enrolled consecutively. We reviewed medical records, including data on postoperative intraocular pressure (IOP) and postoperative complications. The success rate and early postoperative hypertensive phase were compared between groups.

**Results:**

A total of 74 patients with CAGV implantations and 36 patients with FPAGV implantations were studied. The average follow-up periods were 23.3 ± 2.6 months (CAGV) and 22.8 ± 2.8 months (FPAGV; p = 0.424). The surgery time was significantly shorter in the FPAGV group than in the CAGV group (42.6 ± 4.1 vs. 47.3 ± 5.4 min; p < 0.001). Postoperative IOP at 1 week and 1 month were significantly lower in the FPAGV group than in the CAGV group (11.8 ± 3.6 and 14.0 ± 5.3 mmHg vs. 18.7 ± 5.5 and 22.2 ± 5.2 mmHg; p = 0.012 and p = 0.002, respectively). An early postoperative hypertensive phase occurred in 62 eyes, and the frequency was greater in the CAGV group (50 eyes) than the FPAGV group (12 eyes; p = 0.001). There was no significant difference in postoperative complications between the two groups (p = 0.735). The success rate was 84.2% in the FPAGV group and 80.6% in the CAGV group 24 months after surgery (p = 0.367).

**Conclusion:**

FPAGV implantation was associated with a shorter surgery time, without any change in the extent of IOP reduction or complication rate. This procedure may be considered a good alternative for CAGV implantation in patients with refractory glaucoma.

## Introduction

Glaucoma is a leading cause of blindness worldwide [1]. Elevated intraocular pressure (IOP) is the primary risk factor for glaucoma, and reducing IOP is the only proven effective option for treating glaucoma. Medical therapy or laser trabeculoplasty is first tried, and then surgical treatment is considered if these former treatments are not effective at stopping the progression of glaucoma. The firstline surgical option is traditionally trabeculectomy. However, refractory glaucomas such as neovascular glaucoma, secondary glaucoma from uveitis or trauma, and glaucomas with wide conjunctival scars from previous surgeries do not respond well to trabeculectomy [2,3]. Glaucoma tube shunt surgery has been proposed as an alternative approach for patients with refractory glaucoma, and better outcomes from this procedure have been reported [4–6]. Glaucoma drainage device surgery has recently become a widely used therapeutic option for managing glaucoma [7].

The Ahmed glaucoma valve (AGV), a popular glaucoma drainage device, is a useful device for glaucoma tube shunt surgery [8]. In the conventional AGV implantation procedure, a silicone tube is placed into the anterior chamber, the plate is placed between the two adjacent rectus muscles, and the anterior portion of the plate is fixed onto the sclera about 8 mm posterior to the corneoscleral limbus. Fixing the plate on the sclera is a time-consuming procedure and carries risk for complications such as bleeding and perforation of the globe. In addition, in eyes with a narrow palpebral fissure or a small orbit, it may be difficult to obtain a sufficient surgical field to fix the plate.

Some studies have reported the use of sutureless placement in glaucoma drainage surgery. They have shown favorable outcomes without anchoring sutures [9–11]. García-Delpech et al. analyzed the results of 17 patients undergoing full sutureless AGV implantation surgery with a tube tip placed through the pars plana in previously vitrectomized eyes or eyes undergoing combined pars plana vitrectomy [12]. Using this method, they effectively reduced IOP, and there was no extrusion of the tube or plate or plate migration after surgery. However, they did not enroll a control group undergoing conventional AGV implantation in their study, and the study was conducted on patients who underwent vitrectomy at the same time.

To the best of our knowledge, no study has compared surgical outcomes between AGV implantation without plate fixation and conventional AGV implantation with plate fixation. Therefore, in this study we evaluated the safety and efficacy of AGV implantation without plate fixation (free plate AGV [FPAGV]) and compared it with that of conventional AGV (CAGV) implantation surgery with plate fixation.

## Methods

This retrospective study was approved by the institutional review board of Chungnam National University Hospital (CNUH) Institutional Review Board (IRB) (IRB No. 2020-08-070). All procedures were performed in accordance with the tenets of the Declaration of Helsinki. The requirement for informed consent was waived because only data collected during routine outpatient visits was used.

### Subjects

The study enrolled glaucoma patients who underwent conventional AGV (model FP7 with a surface area of 184 mm^2^; New World Medical, Rancho Cucamonga, CA, USA) implantation or free plate AGV implantation at the Department of Ophthalmology, Chungnam National University Hospital, from 2013 to 2017. All patients were followed for more than 1 year after surgery. Neovascular glaucoma, secondary glaucoma resulting from uveitis, ocular trauma or surgery, and glaucoma with a wide conjunctival scar from previous ocular surgery, including failed previous filtration surgery, were included as refractory glaucoma. The surgery was performed if any of the following criteria were met: IOP > 21 mmHg and progression in optic nerve head damage, a defect in the retinal nerve fiber layer, and/or a defect in the visual field despite maximal tolerable medical or laser treatment. Patients < 18 years old, patients who had any other ocular disease except for cataract, and patients who had previously undergone intraocular surgery (except uncomplicated cataract surgery and trabeculectomy) were excluded. Age, sex, presence/absence of systemic disease, history of intraocular surgery or laser treatment, preoperative IOP, number of IOP-lowering medications (a fixed combination agent was counted as two medications), and type of glaucoma were recorded for all patients.

All surgeries were performed by a single experienced surgeon (CSK, who has been performing glaucoma drainage device implantation for >20 years) under retrobulbar anesthesia. All surgeries involved conventional AGV implantation at the beginning of the study period. Both conventional AGV and free plate AGV implantation were performed during the transit period of 2015, and all glaucoma drainage device surgery was changed to free plate AGV by 2016.

**Fig 1:**
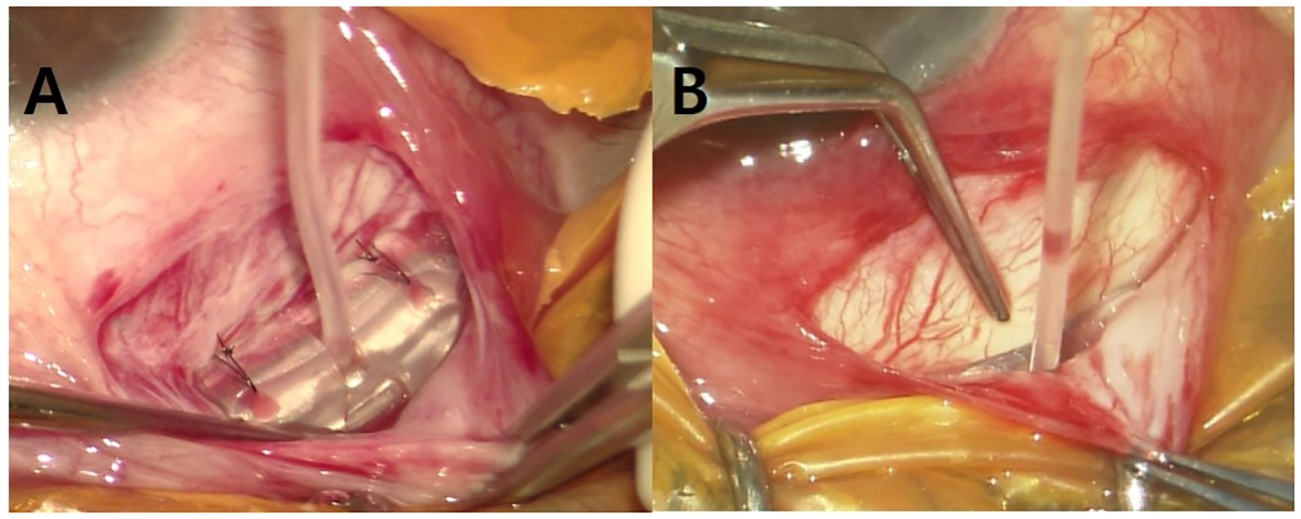
Comparison of conventional Ahmed glaucoma valve (AGV) implantation featuring scleral plate fixation, and free-plate AGV implantation. In conventional AGV implantation, the body of the AGV is fixed on both sides to the sclera at the front edge of the plate (A). Free-plate AGV implantation, does not feature scleral fixation (B).

All AGV implantation was performed on the superotemporal side of the eye. A 8/0 nylon traction suture through the clear cornea was used at the upper peripheral cornea to enhance exposure of the surgical field. All conjunctival flaps were prepared using a limbal-based technique. A 10 mm incision was made to the conjunctiva and Tenon’s capsule, circumferentially 5 mm posterior from the corneal limbus, followed by a dissection between Tenon’s capsule and the sclera. In conventional AGV implantation, the body of the AGV was inserted into the sub-Tenon space and then between the superior and lateral rectus muscles. In conventional AGV implantation, the plate was fixed to the sclera with two 9/0 nylon anchoring sutures at the front edge of the plate on both sides, 8–9 mm from the corneal limbus. In FPAGV implantation, the scleral fixation of the plate was skipped and the center of the plate was located between the superior and lateral rectus muscles (Fig 1). An anterior chamber puncture, parallel to the iris surface, was made about 1 mm posterior to the corneal limbus using a 23 gauge needle. A silicone tube was then cut, and approximately 2 mm of the tube was inserted into the anterior chamber in a bevel-up position. The silicone tube was fixed to the sclera with two anchoring sutures. In the FPAGV procedure, the anterior anchoring suture was fixed onto the sclera with penetration of the silicone tube to avoid possible movement of the tube. The silicone tube near the corneal limbus was covered longitudinally with a 4 × 6 mm full-thickness donor sclera. The Tenon’s capsule and the conjunctiva were sutured with a continuous 10/0 nylon suture. The surgery was completed with a subconjunctival injection of betamethasone.

In all patients, 1% Isopto atropine eye drops (Isopto Atropine; Alcon, Fort Worth, TX, USA) were used for 2 days after the surgery, and 0.3% ofloxacin eye drops (Tarivid; Santen, Osaka, Japan) and 1.0% prednisolone acetate eye drops (Pred Forte; Allergan, Irvine, CA, USA) were used four times a day for 2 weeks and tapered over 6 months.

Postoperative data were collected from patient records for all consecutive visits, and data at 1 week and 1, 3, 6, 12, and 24 months after surgery were analyzed. Postoperative IOP, number of IOP-lowering medications, number of patients who underwent digital ocular massage for elevated IOP, success rate, and number of complications were compared between the CAGV and FPAGV groups. In addition, we evaluated the early postoperative hypertensive phase in both groups. In our study, the early hypertensive phase was defined as postoperative IOP > 21 mmHg within the first 3 months [13]. We also evaluated qualified and complete success in both groups. Success was defined as IOP = 6–21 mmHg 1 month after surgery and >20% reduction from baseline; a complete success was defined as meeting these criteria without the use of IOP-lowering medications. The surgery time was determined from the time of surgical incision to the time of final subconjunctival injection using the surgical record of the digital medical chart and was compared between groups.

### Statistical analyses

PASW version 18.0 (SPSS, Chicago, IL, USA) was used for statistical analyses of all data. Independent t tests and chi-square tests were used to compare clinical characteristics, number of digital ocular massages, surgery time, and postoperative hypertensive phase between the CAGV and FPAGV implantation groups. We used independent t tests to compare IOP and the number of medications at each visit between the two groups. Paired t tests were used to compare IOP and the number of medications at each visit with baselines. Fisher’s exact tests were used to compare postoperative complications between the two groups. Kaplan–Meier survival analyses were performed to assess the frequency of surgical success after AGV implantation. P < 0.05 was considered statistically significant.

## Results

The records for 110 eyes (of 110 patients) were examined. There were 74 CAGV implantation patients (48 males and 26 females) and 36 FPAGV implantation patients (20 males and 16 females). The mean ages of the two groups were 62.4 ± 13.8 years (CAGV) and 61.8 ± 14.1 years (FPAGV; p = 0.832). The average follow-up period of the CAGV group was 23.3 ± 2.6 months, and 68 eyes were followed-up for 24 months. The average follow-up period of the FPAGV group was 22.8 ± 2.8 months, and 32 eyes were followed-up for 24 months (p = 0.424). There were no significant differences between the two groups in sex, systemic disease, axial length, corneal endothelial cell density, or previous interventions including trabeculectomy (all ps > 0.05; Table 1). Surgery time was significantly shorter in the FPAGV group than in the CAGV group (42.6 ± 4.1 vs. 47.3 ± 5.4 min; p < 0.001).

**Table 1:** Clinical characteristics of the study patients.

	CAGV group	FPAGV group	p value
No. of patients (no. of eyes)	74 (74)	36 (36)	
Age (years, mean ± SD)*	62.4 ± 13.8	61.8 ± 14.1	0.832
Sex (M/F)†	48/26	20/16	0.346
Mean follow-up period (months)	23.3 ± 2.6	22.8 ± 2.8	0.424
Systemic disease, n (%)†			
DM	21 (28.4)	15 (41.7)	0.163
HTN	27 (36.5)	14 (38.9)	0.807
Axial length (mm)*	24.4 ± 1.8	24.6 ± 1.6	0.576
Corneal endothelial cell density (cells/mm2)*	2051.9 ± 686.0	1965.8 ± 755.8	0.560
Previous interventions, n (%)†			
Laser trabeculoplasty	5 (6.8)	4 (11.1)	0.434
Trabeculectomy	21 (28.4)	15 (41.7)	0.163
Diagnosis, n (%)†			0.293
Primary open-angle glaucoma	22 (29.7)	16 (44.4)	
Uveitic glaucoma	26 (35.1)	11 (30.6)	
Neovascular glaucoma	8 (10.8)	5 (13.9)	
Chronic angle closure glaucoma	5 (6.8)	0 (0)	
Other secondary glaucoma	13 (17.6)	4 (11.1)	
Operation times (minutes)*	47.3 ± 5.4	42.6 ± 4.1	<0.001

Data are the mean ± SD.

*Independent t-test,

^†^Chi-square test.

CAGV = conventional Ahmed glaucoma valve; FPAGV = free plate Ahmed glaucoma valve; SD = standard deviation.

Preoperative IOP was 31.7 ± 9.4 mmHg in the CAGV group and 31.8 ± 7.8 mmHg in the FPAGV group, with no significant difference between the groups (p = 0.984). In both groups, IOP was significantly lower at all postoperative visits than at baseline (all ps < 0.05). Postoperative IOP at 1 week was significantly lower in the FPAGV group than in the CAGV group (11.8 ± 3.6 vs. 14.0 ± 5.3 mmHg, respectively; p = 0.012). In addition, postoperative IOP at 1 month was significantly lower in the FPAGV group than in the CAGV group (18.7 ± 5.5 vs. 22.2 ± 5.2 mmHg, respectively; p = 0.002). There were no significant differences in postoperative IOP between the two groups at 3, 6, 12, or 24 months (all ps > 0.05; Table 2).

**Table 2:** Comparison of intraocular pressure and number of anti-glaucoma medications.

	IOP (mm Hg, mean ± SD)				No. of medications (mean ± SD)			
	No	CAGV group	FPAGV group	p value*	No	CAGV group	FPAGV group	p value*
Baseline	74	31.7 ± 9.4	31.8 ± 7.8	0.984	36	3.8 ± 0.4	3.7 ± 0.4	0.497
After surgery								
1 week	74	14.0 ± 5.3†	11.8 ± 3.6†	0.012	36	0.0 ± 0.0†	0.0 ± 0.0†	-
1 month	74	22.2 ± 5.2†	18.7 ± 5.5†	0.002	36	0.4 ± 0.8†	0.2 ± 0.7†	0.344
3 months	74	18.3 ± 4.5†	17.5 ± 5.6†	0.429	36	1.9 ± 0.8†	1.5 ± 1.1†	0.100
6 months	74	17.0 ± 4.0†	17.2 ± 5.2†	0.807	36	2.0 ± 1.0†	1.8 ± 1.2†	0.354
12 months	74	16.5 ± 3.4†	15.8 ± 3.8†	0.320	36	2.1 ± 1.1†	2.0 ± 1.2†	0.488
24 months	68	16.4 ± 3.8†	15.7 ± 4.8†	0.437	32	2.3 ± 1.1†	2.1 ± 1.2†	0.684

Data are the mean ± SD.

* Independent t-test,

^†^ Significantly lower than baseline at all time interval (p < 0.05, paired t-test).

IOP = intraocular pressure; CAGV = conventional Ahmed glaucoma valve; FPAGV = free plate Ahmed glaucoma valve; SD = standard deviation.

The number of preoperative IOP-lowering medications was 3.8 ± 0.4 in the CAGV group and 3.7 ± 0.4 in the FPAGV group, with no significant difference between the groups (p = 0.497). The number of medications was significantly lower at all postoperative visits than at baseline (all ps < 0.05). At postoperative 1 week, no patient used an IOP-lowering medication. There were no significant differences in the number of medications between the two groups at 1, 3, 6, 12, or 24 months (all ps > 0.05; Table 2).

Postoperative complications occurred in 18 patients (24.3%) in the CAGV group and 11 patients (30.6%) in the FPAGV group (p = 0.735). Postoperative complications lasting more than a week or needing additional intervention included the tube-iris touch, tube occlusion, shallow anterior chamber, hypotony (IOP < 6 mmHg), choroidal effusion, corneal edema (focal and diffuse), diplopia, and hyphema. Plate or tube exposure or migration were not observed in either group. The frequencies of any of the postoperative complications did not differ significantly between the two groups (all ps > 0.05; Table 3). For the significant shallow anterior chamber, which developed in two eyes of the CAGV group and one eye of the FPAGV group, a viscoelastic material (Healon GV; Advanced Medical Optics, Santa Ana, CA, USA) was injected into the anterior chamber an average of 1.7 times, and the anterior chamber was stabilized within 1 month without further management in all cases. Tube occlusion developed in five eyes (four in the CAGV group and one in the FPAGV group), and a neodymium YAG (Nd:YAG) laser in two patients and surgical iridectomy in one patient were needed to resolve the occlusion of the tube. Diplopia developed in seven eyes (five in the CAGV group and two in the FPAGV group); Fresnel prism glasses were prescribed to reduce the discomfort in one case, and the AGV was removed in one case of intractable significant diplopia.

**Table 3:** Postoperative complications.

	CAGV group (n = 74)	FPAGV group (n = 36)	p value*
Tube-corneal touch	0 (0.0)	0 (0.0)	
Tube-iris touch	1 (1.4)	0 (0.0)	0.743
Tube occlusion	4 (5.4)†	1 (2.8)	0.486
Plate or tube exposure	0 (0.0)	0 (0.0)	-
Plate or tube migration	0 (0.0)	0 (0.0)	-
Shallow anterior chamber‡	2 (2.7)	1 (2.8)	0.841
Hypotony (IOP < 6 mmHg)	2 (2.7)	2 (5.6)	0.596
Choroidal effusion	1 (1.4)	1 (2.8)	0.549
Corneal edema			
Focal	2 (2.7)	2 (5.6)	0.596
Diffuse	5 (6.8)	2 (5.6)	0.641
Diplopia§	5 (6.8)	2 (5.6)	0.641
Hyphema	1 (1.4)	1 (2.8)	0.549
Endophthalmitis	0 (0.0)	0 (0.0)	-
No. of patient with complication∥	18 (24.3)	11 (30.6)	0.735

Values are presented as n (%).

*Fisher’s exact test.

^†^There were 3 patients of tube occlusion treated with Nd:YAG (2 patients) and iridectomy (1 patient).

^‡^All patients with shallow anterior chamber (3 patients, 2.7%) needed viscoelastic device injection.

^§^One patient with diplopia undergone device removal 2 years later.

^∥^Chi-square test.

CAGV = conventional Ahmed glaucoma valve; FPAGV = free plate Ahmed glaucoma valve; Nd:YAG = neodymium doped yttrium aluminum garnet.

**Fig 2:**
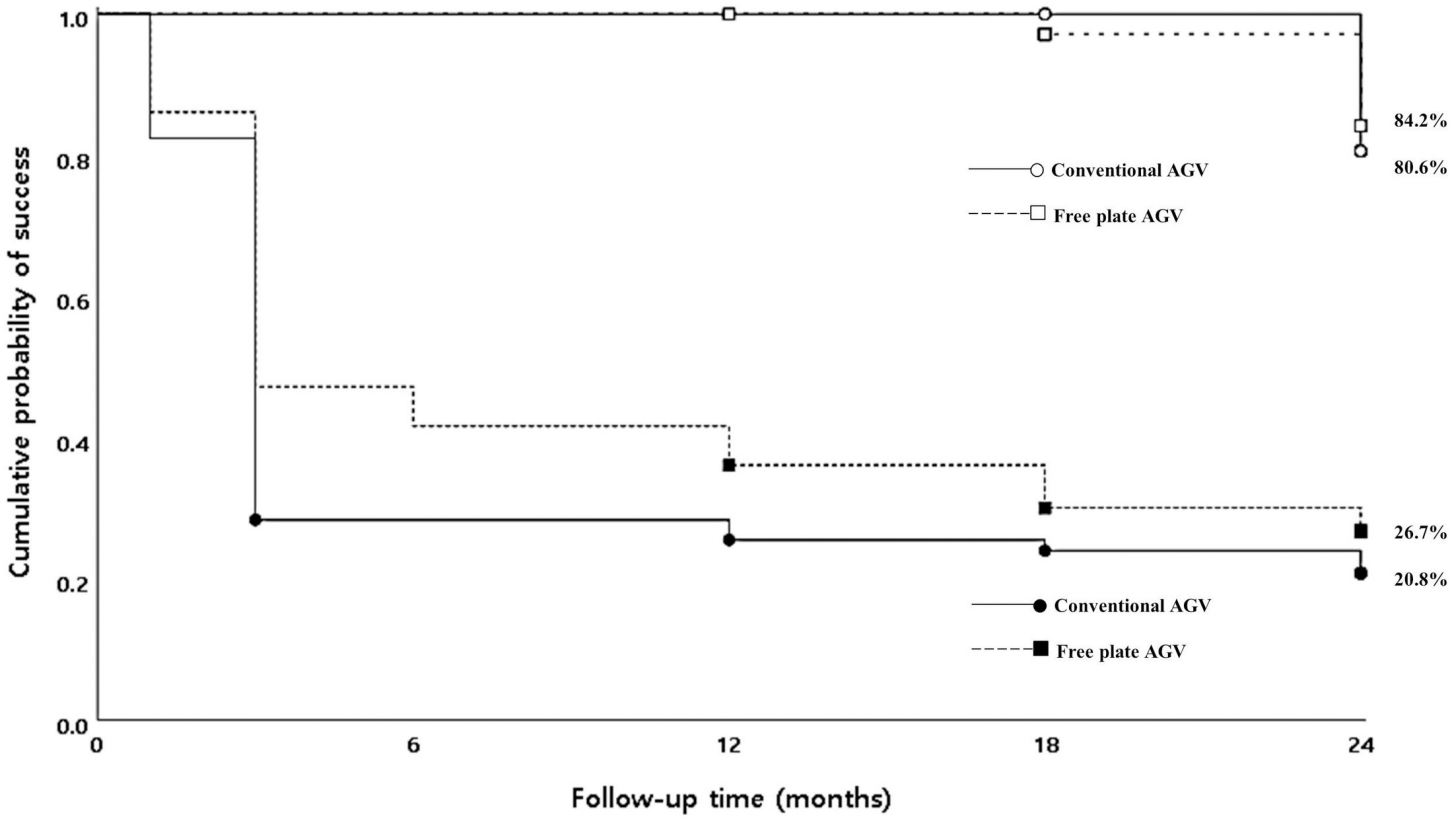
Kaplan–Meier survival curves showing the success rates of the conventional Ahmed glaucoma valve (CAGV) group and the free-plate Ahmed glaucoma valve (FPAGV) group. The survival rates were 80.6% in the CAGV group and 84.2% in the FPAGV group at 24 months (p = 0.367). The complete success rate were 20.8% in the CAGV group and 26.7% in the FPAGV group at 24 months (p = 0.354).

An early postoperative hypertensive phase occurred in 62 eyes; the frequency was greater in the CAGV group (50 eyes, 67.6%) than the FPAGV group (12 eyes, 33.3%; p = 0.001). In addition, the interval between surgery and onset of a hypertensive phase was greater in the FPAGV group than in the CAGV group (1.33 ± 0.75 vs. 1.24 ± 0.66 months, respectively; p = 0.002). Elevated IOP was treated with a beta blocker, a fixed combination of beta blockers, a carbonic anhydrase inhibitor, and prostaglandin analogues as needed, along with digital massage. The number of patients who underwent digital ocular massage to control elevated IOP was 45 (60.8%) in the CAGV group and 19 (52.8%) in the FPAGV group, with no significant difference between the groups (p = 0.423). Cumulative Kaplan–Meier survival analysis results for the success of surgery, defined by postoperative reduction in IOP, were 84.2% in the FPAGV group and 80.6% in the CAGV group 24 months after surgery. There was no significant difference in success between the two groups (p = 0.367). Complete success was 20.8% in the CAGV group and 26.7% in the FPAGV group, with no significant difference between the groups (p = 0.354; Fig 2).

## Discussion

Glaucoma tube shunt surgery is useful for treating refractory glaucoma and is widely used [14–16]. Among glaucoma drainage devices, AGV is widely used because it is easy to insert during surgery, has a wide filtration area, and has a valve function to prevent hypotony due to excessive drainage of the aqueous [17,18]. However, all glaucoma drainage devices that use a postlimbal plate cause tissue trauma while the plate is being inserted and fixed to the sclera with sutures. Particularly in an eye with a small palpebral fissure, orbit, or axial length, which is relatively common in Asians, it may be difficult to obtain a good surgical field view for the fixation process; thus, glaucoma drainage surgery can be difficult or limited in some cases.

If fixation of the plate could be avoided, and the safety and efficacy of the surgery were identical to those of conventional insertion techniques, it may be easier and save time. García-Delpech et al. developed a sutureless valve implantation technique to treat refractory glaucoma. The IOP was significantly reduced after the procedure, and the safety was comparable to that of other glaucoma surgeries [12]. Pham et al. performed sutureless AGV implantation using a pericardial patch graft instead of sutures, either alone or combined with other operations. The IOP reduction, number of medications required, and complication rate were similar to those of previous studies on tube shunt implantation [19]. In our study, the postoperative IOPs at 2 years were 16.4 ± 3.8 and 15.7 ± 4.8 mmHg in the CAGV and FPAGV groups, respectively. Both groups showed a significant reduction in IOP compared to baseline IOP, and reductions in IOP did not differ significantly in the two groups during the study period, except for the early postoperative period. The complication frequencies also did not significantly differ, similar to the results of previous multicenter study [20,21].

An increase in IOP in the early phase after surgery is a frequent finding after glaucoma shunt surgery. It has been reported after the implantation of many types of glaucoma drainage devices, including Baerveldt, single- and double-plate Molteno implants, and Krupin valves [22–24]. An increase in IOP was also observed after AGV implantation [25,26]. Ayyala and colleagues reported a so-called hypertensive phase (HP) in 70 patients (82%) who underwent insertion of an AGV to control refractory glaucoma [25]. This phase may be related to inflammation and the emergence of a dense layer of fibrous tissue over the plate [18,22]. Nouri-Mahdavi and Caprioli suggested that the occurrence of an HP predicted a less desirable tonometry outcome 6–12 months after surgery, so the development of an HP is an unfavorable prognostic sign [13,27]. In our study, the hypertensive phase was defined as an IOP > 21 mmHg within the first 3 months after surgery. An HP occurred more frequently in the CAGV group than the FPAGV group (50 eyes, 67.6% vs. 12 eyes, 33.3%, respectively; p = 0.001). Postoperative IOP at 1 month was lower in the FPAGV group than the CAGV group (18.7 ± 5.5 vs. 22.2 ± 5.2 mmHg, respectively; p = 0.002). Because the plate was not fixed onto the sclera in the FPAGV group, we assume that there might have been less chance of bleeding, tissue trauma, and resultant inflammation; thus, capsule formation around the plate was accomplished in a more favorable environment in the early postoperative period in the FPAGV group compared to the CAGV group. In addition, the plate was not closely attached to the sclera with sutures, which suggests that capsule formation may form both above and below the plate. This observation anticipates that the aqueous reservoir around the plate may be larger and wider in the FPAGV group. In our study, the FPAGV group showed greater success than the CAGV group 2 years after the surgery, although this difference was not statistically significant.

**Fig 3:**
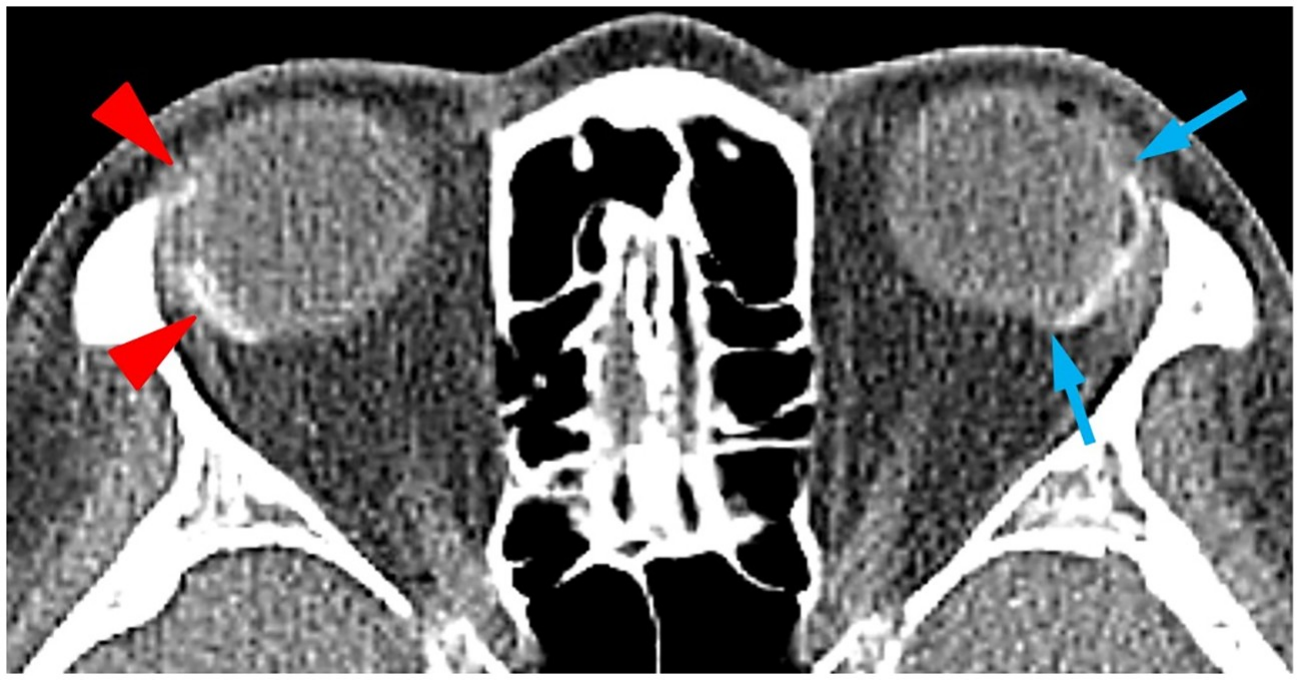
A facial computed tomography image of a patient who underwent Ahmed glaucoma valve (AGV) implantation in both eyes. In our study, one patient underwent two separate implantations in either eye. Initially conventional AGV implantation was performed on the right eye (arrowhead) and free-plate AGV implantation performed on the left eye (arrow) 3 years later. One year after the final surgery, we obtained a facial computed tomography scan to verify implant locations; both plates were safely positioned (well posterior to the equator) without any notable difference in location.

Postoperative complications occurred in 23 eyes (31.1%) in the CAGV group and 12 eyes (33.3%) in the FPAGV group. These results are similar to those reported in previous studies [20,21]. Many complications, such as tube-iris touch, hypotony, and hyphema, were transient and self-limiting. Patients with a tube occlusion or shallow anterior chamber required minor intervention with a Nd:YAG laser, iridectomy, or viscoelastic device injection. The most common complication was corneal edema (focal or diffuse), which affected seven eyes (9.5%) of the CAGV group and four eyes (11.2%) of the FPAGV group. Most patients with corneal edema had a low preoperative corneal endothelial cell density. None of these edemas progressed, and no patient required corneal transplantation during the study period. There were two eyes (2.7%) with hypotony in the CAGV group and two eyes (5.6%) with hypotony in the FPAGV group. In all patients, IOP was stabilized without additional management within 4 weeks. Law et al. hypothesized that dynamic movement of the plate could potentially occur due to separation of the plate from the sclera because of suture loosening [28]. However, there was no case of plate or tube exposure or migration during the 2 years of follow-up (Fig 3). Although we did not fix the plate to the sclera with sutures, a secure tube fixation penetrating the tube using 10/0 nylon onto the sclera and covering of the tube by a full thickness donor sclera in almost the entire path to the plate could have securely fixed the implant during the encapsulation process. We believe that if the plate can be kept in a secure position until the primary encapsulation process is completed and the fibrous tissue penetrates through the holes in the plate body about 4–8 weeks after surgery, there is almost no chance of movement of the plate after surgery.

The surgery time for FPAGV implantation was 42.6 ± 4.1 min, which was shorter than the 47.3 ± 5.4 min for CAGV implantation (p < 0.001). A 5 min difference may not seem to be a big difference when the whole procedure is more than 40 min. However, given that the other surgical steps are essentially identical, any reduction in surgery time is useful in clinical practice.

There are some limitations to our study. First, this was a retrospective study, so we could not use a randomized control group for comparison. CAGV was performed in all cases in the first half of the study period, and FPAGV was mainly performed in the last half of the study. However, all surgeries were performed by a single surgeon very experienced in glaucoma drainage implant surgery; therefore, we do not think that there was a significant difference in surgical technique or learning curve in the surgical procedure. Second, we did not analyze the aqueous reservoir formed around the plate in the early postoperative period using an imaging test such as anterior segment optical coherence tomography, so further prospective studies are needed to evaluate the aqueous reservoir in both groups through imaging. Third, the success and complications of the surgery would be better investigated using a larger number of patients for a longer period of follow-up undergoing both types of AGV implantation at the same time. Finally, all glaucoma tube shunts were of the AGV (FP7) type; our observations may not apply to other glaucoma drainage devices or other type of AGVs.

In conclusion, free plate Ahmed valve implantation had some advantages. It was faster and easier than conventional implantation, which required fixation of the plate to the sclera. Using this technique, we avoided some possible complications associated with fixation, such as bleeding or perforation of the globe, and also reduced tissue trauma, because we did not need to obtain a good surgical field view for the fixation process. In addition, there were no significant differences between groups in IOP reduction or the incidence of surgical complications. Based on these results, we conclude that the efficacy and safety of FPAGV implantation are comparable to those of conventional AGV implantation with plate fixation, and FPAGV is considered a good alternative to conventional surgical techniques.

## Supporting information

S1 Data (XLSX)
